# An overview of human prion diseases

**DOI:** 10.1186/1743-422X-8-559

**Published:** 2011-12-24

**Authors:** Muhammad Imran, Saqib Mahmood

**Affiliations:** 1 Department of Physiology and Cell Biology, Centre for Research in Endocrinology and Reproductive Sciences (CRERS), University of Health Sciences (UHS), Khayaban-e-Jamia Punjab, Lahore 54600, Pakistan; 2 Department of Human Genetics and Molecular Biology, University of Health Sciences (UHS), Khayaban-e-Jamia Punjab, Lahore 54600, Pakistan

**Keywords:** Prions, Prion diseases, *PRNP*, TSE, CJD

## Abstract

Prion diseases are transmissible, progressive and invariably fatal neurodegenerative conditions associated with misfolding and aggregation of a host-encoded cellular prion protein, PrP^C^. They have occurred in a wide range of mammalian species including human. Human prion diseases can arise sporadically, be hereditary or be acquired. Sporadic human prion diseases include Cruetzfeldt-Jacob disease (CJD), fatal insomnia and variably protease-sensitive prionopathy. Genetic or familial prion diseases are caused by autosomal dominantly inherited mutations in the gene encoding for PrP^C ^and include familial or genetic CJD, fatal familial insomnia and Gerstmann-Sträussler-Scheinker syndrome. Acquired human prion diseases account for only 5% of cases of human prion disease. They include kuru, iatrogenic CJD and a new variant form of CJD that was transmitted to humans from affected cattle via meat consumption especially brain. This review presents information on the epidemiology, etiology, clinical assessment, neuropathology and public health concerns of human prion diseases. The role of the PrP encoding gene (*PRNP*) in conferring susceptibility to human prion diseases is also discussed.

## Background

Prion diseases are transmissible protein misfolding disorders in which misfolding of a host-encoded prion protein (PrP) occurs. PrP is a 253 amino acids (aa) protein. The first 22 N-terminal aa are removed from PrP after its transport to endoplasmic reticulum, while the last 23 C-terminal aa are cleaved off after the addition of glycosylphosphatidylinositol (GPI) anchor, which helps the protein to attach to the outer surface of cell membranes. PrP may exist in two forms: a normal cellular prion protein designated as PrP^C ^and a pathogenic misfolded conformer designated as PrP^Sc^. Both PrP^C ^and PrP^Sc ^conformers are encoded from the same sequence of the 16 kb single copy *PRNP *gene that is positioned on the short (p) arm of human chromosome 20 (20p13), from base pairs 4,666,796-4,682,233. The human *PRNP *contains two exons with the second one carrying the whole open reading frame. The abnormal PrP^Sc ^isoform differs from the normal PrP^C ^isoform in secondary and tertiary structure, but not in primary amino acids sequence. PrP^C ^is predominantly rich in alpha helical contents, while PrP^Sc ^is predominantly rich in beta sheet contents [1-5]. This conformational discrepancy renders the PrP^Sc ^isoform extremely resistant to proteolysis and degradation by conventional means of chemical and physical decontamination or disinfection. In contrast to PrP^Sc^, PrP^C ^is soluble in non-denaturing detergents and is completely degraded by proteases [1,3].

The superscript (Sc) has been used to refer to scrapie, the first and the most ancient animal Transmissible Spongiform Encephalopathy (TSE). Many authors also use superscripts other than (Sc) to distinguish normal and pathogenic (disease-causing) isoforms. These include (res) for resistant and (Dis) for disease. An abbreviated name of a prion disease can also be used as superscript to point out the origin of the pathogenic isoform i.e. PrP^Sc ^or PrP^CJD^. The pathogenic conformers are simply called prions (the infectious protein particles) [3].

According to seeding-nucleation model, the preexisting or acquired PrP^Sc ^oligomers catalyze the conversion of PrP^C ^molecules into PrP^Sc ^fibrils the breakage of which provides more PrP^Sc ^templates for the conversion process. The process of prion propagation in the brain results in the pathogenesis of prion diseases [6]. Sixteen different variants of prion disease have been reported so far: nine in humans and seven in animals. The etiology, host range and year of description for these disease variants are given in Table 1. In the present review, a brief description of human prion diseases is provided.

**Table 1:** Etiology of prion disease

Animal prion diseases				
**Disease**	**Host**	**Etiology**	**Year of Description**	**References**

Scrapie	Sheep, Goats	Infection with Prions of unknown origin	Mid 18th century	[7,8]

TME	Mink	Infection with Prions of either sheep or cattle origin	1947	[8-10]

CWD	Cervids	Infection with Prions of unknown origin	1967	[8,10,11]

BSE	Cattle	Infection with Prions of unknown origin	1986	[8,12]

EUE	Nyala, Kudu	Infection with Prions of BSE origin	1986	[8,10,13,14]

FSE	Cats	Infection with Prions of BSE origin	1990	[8,10,15]

NHP	Lemurs	Infection with Prions of BSE origin	1996	[8,10,16]

**Human prion diseases**				

**Disease**	**Host**	**Etiology**	**Year of Description**	**References**

Kuru	Human	Ritualistic Cannibalism or "Transumption"	1957	[17]

sCJD	Human	Spontaneous PrP^C^→PrP^Sc ^conversion or somatic mutation	1920	[18,19]

f/gCJD	Human	Mutations in *PRNP*	1924	[20]

GSS	Human	Mutations in *PRNP*	1936	[21]

iCJD	Human	Infection with Prions of human origin by cadaveric corneal grafts, hGH or dura mater	1974	[22]

FFI	Human	*PRNP *haplotype 178N-129M	1986	[23]

vCJD	Human	Infection with Prions of BSE origin	1996	[24]

sFI	Human	Spontaneous PrP^C^→PrP^Sc ^conversion or somatic mutation	1999	[25]

VPSPr	Human	Spontaneous PrP^C^→PrP^Sc ^conversion or somatic mutation	2008	[26]

### Sporadic Cruetzfeldt-Jacob disease (sCJD)

Sporadic Cruetzfeldt-Jacob disease accounts for 85% of all CJD cases with annual worldwide incidence of 1-2 cases/million population [27]. It occurs equally in both sexes with a peak age of onset between 55 and 75 years. Some younger (below 20 years) and oldest (above 90 years) cases have also been reported. Clinical symptoms include rapidly progressive dementia, cerebellar dysfunction including muscle incoordination, and visual, speech and gait abnormalities. Dementia is the major symptom followed by spontaneous or induced myoclonia. During the disease course, symptoms of pyramidal and extrapyramidal dysfunction with reflexes, tremors, spasticity and rigidity, and behavioral changes with agitation, confusion and depression may also be observed. At the end of the disease course, most of patients go into a state of akinetic mutism (they become unresponsive to exterior stimuli) [28,29].

The presence of spongiform changes and sparse PrP^Sc ^distribution in CNS of sCJD patients are the hallmark of neuropathology of the disease. The deposits of amyloid plaques may also be noticed in 5-10% of cases [28,30]. On the basis of clinicopathological findings, different variants of sCJD have been described. Amaurotic or Heindenhain variant is characterized by rapidly progressive dementia, myoclonus, visual disturbances such as hallucinations, visual agnosia and cortical blindness, and short disease duration; Brownell and Oppenheimer variant is characterized by an early and predominant cerebellar ataxia and relatively late dementia; the thalamic form is characterized by dementia and movement disorders; while the panencephalic Japanese variant is characterized by high amount of cells in CSF, profound damage of the white matter and a very slow disease course [28,29,31,32].

On the basis of clinical, biological, electrophysiological and neuropathological findings, sCJD cases can be classified as possible, probable or definite cases. Possible cases are characterized by rapidly progressive dementia accompanied by at least two of the following symptoms: myoclonus, cerebellar or visual symptoms, pyramidal or extrapyramidal signs, akinetic mutism and disease course of less than 2 years. Possible cases are considered probable cases when they reveal a characteristic periodic EEG or increased levels of 14-3-3 protein in CSF [29]. Definite cases are defined by the presence of spongiform changes or PrP^Sc ^reactivity in brain. Notably, sCJD symptoms may also be seen in disorders like Alzheimer's disease, diffuse Lewy body disease or frontal dementia, paraneoplastic syndrome, tumor or stroke and in disorders with toxic, nutrition, metabolism or infection-related causes. Differential diagnosis for possible and probable cases of sCJD is, therefore, needed. Computed tomography (CT) may reveal signal of mild brain atrophy in some cases. The use of cranial magnetic resonance imaging (MRI) is helpful in detecting brain atrophy and, in more than 50% of cases, may reveal high signals on T2, flair or diffusion-weighted sequences in basal ganglia or in cerebral or cerebellar cortex [29]. Almost all sCJD cases show altered electroencephalographic (EEG) signals. Slow wave activity remains constant and deteriorates with the progression of the disease. Pseudo-periodic sharp wave complexes of 1 Hertz are characteristic of EEG in sCJD cases. To overcome the transient nature of periodic sharp wave complexes, repeated EEG recordings are required [29,33].

The clinicopathological and molecular phenotypes of sCJD are greatly influenced by variations in *PRNP*. *PRNP *polymorphisms in regulatory as well as coding sequences have been associated with predisposition to the disease development [34-36]. Homozygosity at M129V polymorphism is a strong risk factor for the development of sCJD [36,37]. In Japanese, homozygosity at another *PRNP *polymorphism E219K is also a risk factor for the development of sCJD [38,39]. E219K has been found in only Asian and Pacific populations which are more homozygous for methionone at codon 129 as compared to Western populations [27,40]. Inefficient interaction between heterologous PrP^C ^molecules encoded from distinct *PRNP *alleles (129 M and 129 V) may restrict the conversion of PrP^C ^into PrP^Sc^. Any constraint in the process of prion propagation is assumed to slow down the progression of the disease [36]. The clinical phenotypic heterogeneity seen in sCJD cases can be attributed to different prion strains. A strain is biochemically defined by pattern and ratios of PrP^Sc ^fragments on Western blot. Three M129V genotypes (129 MM, 129 MV and 129 VV) and 2 strain types (type 1 and type 2) can be coupled into 6 combinations (MM1, MV1, VV1, MM2, MV2 and VV2) which correspond very well to different clinical phenotypes of sCJD. On the basis of these 6 combinations, three different strain classification systems have been proposed [32,41-43].

### Fatal familial insomnia (FFI)

Previously known as thalamic dementia but renamed in 1986 as fatal familial insomnia (FFI) is an autosomal dominantly inherited human prion disease. FFI is caused by a *PRNP *mutation D178N linked to methionine of the *PRNP *polymorphism M129V. When linked to valine at the codon 129, the same mutation leads to a diverse phenotype of f/gCJD [23,44,45]. So far, almost 100 cases of FFI in almost 40 families have been reported from Italy, Germany, Austria, Spain, UK, France, Finland, the United States, Australia, Japan, China, and Morocco [46].

FFI occurs equally in men and women without any significant difference between 129 MM and 129 MV genotypes. However, the disease duration may be significantly shorter in 129 MM subjects. The FFI cases present between 20 and 72 years with an average of 49 years and may live after the disease onset for 8-72 months with an average of 18.4 months. Clinical symptoms include insomnia or disrupted sleep. Myoclonus, ataxia, dysarthria, dysphagia, and pyramidal signs, and autonomic hyperactivation can also be noted. Severity and sequence of clinical symptoms may differ between 129 MM and 129 MV genotypes. Insomnia, myoclonus and autonomic dysfunction are often more severe in 129 MM subjects, while ataxia, dysarthria and seizures often predominate in 129 MV subjects [28,29,44,45,47].

Polysomnography is very useful for the diagnosis of the disease. It provides evidence of insomnia by displaying marked reduction in sleep time and disorderly transition between sleep stages. The 129 MV genotype is known to reduce the severity of these signs of abnormal sleep. In contrast, the 129 MV genotype is associated with more widespread hypometabolism in the thalamus and cingulate cortex as revealed by positron emission tomography (PET). Thus, PET may be another useful tool for the diagnosis of FFI. With later cerebral cortical and cerebellar involvement, neuronal loss and astrogliosis occur predominantly in the anterior and dorsomedian thalamic nuclei in all cases and in the inferior olives in most cases with scant PrP deposition. However, PrP deposits may be more evident in the molecular layer of the cerebellum with a strip like pattern and in the subiculum entorhinal region [28,29,44,45,47].

### Sporadic fatal insomnia (sFI)

A sporadic form of fatal insomnia (sFI) was reported in 1999. The patients showed clinical and neuropathological signs similar to FFI but family history and mutations in *PRNP *were not evident. All patients were 129 MM homozygotes and biochemical strain typing of these disease subtypes revealed the only difference in the intensity of the unglycosylated fragment of PrP^Sc ^type 2; the unglycosylated fragment was under-represented in FFI [25,45,48]. So far, near 24 sFI cases all revealing 129 MM homozygosity and propagating PrP^Sc ^type 2 have been reported in various countries of the world [49]. sFI is occasionally also referred to as "sCJDMM2 thalamic" but is clinicopathologically sufficiently distinct from sCJDMM2. In sCJDMM2, cerebral cortex is most severely affected rather than thalamus where possibly the circadian rhythms of sleep-wake cycle are controlled [42,45]. How the same MM2 PrP^Sc ^encodes distinct phenotypes in sCJD and sFI is a matter of concern, indicating that factors additional to the codon 129 genotype and PrP^Sc ^type may also be involved in the determination of prion strains. However, MM2 PrP^Sc ^conformers associated with sCJD and sFI may show variation in the intensity and size of their fragments by the application of more sensitive 2-dimensional electrophoretic separation and improved PK digestion conditions [42].

### Familial or genetic CJD (f/gCJD)

Familial CJD (fCJD) is associated with dominantly inherited point mutations in *PRNP*. There is a region of 5 repeats within PrP from residues 51-91 comprised of a nonapeptide PQGGGGWGQ and four tandem octapeptide repeats (PHGGGWGQ)_4_. Insertions of 2-9 octapeptide repeats (2-9-OPRI) and a deletion of 2 octapeptide repeats (2-OPRD) in *PRNP *are also associated with susceptibility to fCJD. Because more than 50% of fCJD cases have been reported without a positive family history of the disease, the term "genetic CJD (gCJD)" is now being used more frequently instead of fCJD. There is much variation in the penetrance of different mutations as evidenced by considerable intrafamilial and intragenerational phenotypic variability; sometimes, the disease may skip a generation. The incidence of gCJD is 5-15% of all CJD cases. Although the familial nature of gCJD is known since 1920s, mutations associated with the disease were first reported in 1989 only 2 years after the cloning of *PRNP *cDNA [28,29,42,44,47,50]. The frequency of mutations differs worldwide; however, geographical or ethnic clusters of gCJD cases with mutations E200K, I210V, D178N, and V180I have been reported from Israel, Slovakia, Chile, Italy, Spain and Japan [42,51].

Similar to CJD, the clinicopathological phenotype in gCJD largely correlates with the codon 129 genotypes and PrP^Sc ^type, and mutations appear to play a role not more than a susceptibility factor. Whether *PRNP *polymorphisms other than M129V also influence the disease characteristics is unknown. Multiple strains, although yet remain unidentified, may be involved in the pathogenesis of the disease. For some gCJD cases, fragments of 12-18.5 kDa in addition to fragments of 19 and 21 kDa which characterize PrP^Sc ^types 2 and 1, respectively, can also be found. *PRNP *mutations may play their part in the pathogenesis of gCJD by i) increasing the propensity of PrP^C ^and PrP^M ^for aggregation, ii) increasing the retention of aggregated PrP molecules within secretory pathways, and iii) modulating the intramolecular salt-bridge and hydrogen bond interactions. Disturbance in intramolecular interactions is known to project the hydrophobic amino acids outside making the protein insoluble [44]. With increase of every OPR, both the ability of PrP^M ^for binding divalent ions such as copper and the severity of clinicopathological phenotype are increased. The biochemical strain typing of gCJD often reveals the underrepresentation of unglycosylated fragments. This is because unglycosylated fragments are more unstable than mono- and diglycosylated fragments and are, therefore, more inefficient in their transport from secretory pathways to the plasma membrane [42,44,52]. Although clinical phenotypes related to most of mutations overlap with those of sCJD [42], the clinical phenotype in gCJD cases carrying the T183A-129M haplotype is slightly distinctive and incorporates personality changes followed by rapidly progressive dementia, with fronto-temporal features such as aggressive behavior, hyperorality, verbal stereotypes and often parkinsonian signs [53].

### Gerstmann-Sträussler-Scheinker syndrome (GSS)

Autosomal dominant inheritance of *PRNP *mutations may also lead to another form of human TSE named as Gerstmann-Sträussler-Scheinker Syndrome (GSS). The incidence of GSS is 1 in 100 million of population per year [28]. GSS is characterized by an early onset between 30 and 60 years of age and slow disease progression extended over a period of 3.5-9.5 years. Clinical symptoms which may show intrafamilial and intragenerational variation include cerebellar ataxia, gait abnormalities, dementia, dysarthria, ocular dysmetria, infrequent myoclonus, spastic paraparesis, parkinsonian signs, and hyporeflexia or areflexia in the lower extremities [28,29]. However, legs hyperreflexia from an early disease course has recently been seen in a Japanese GSS patient carrying the P102L mutation [54]. In GSS, altered sleep and temperature rhythms may also be noticed [55]. The *PRNP *polymorphism M129V has been shown to influence the disease phenotype. When linked to V129 allele, P102L, which is the most common cause of GSS, resulted in the predominance of psychiatric signs such as apathy and depression [56]. EEG is rarely diagnostic in GSS. Following *PRNP *mutations have been shown to cosegregate with GSS: P102L, P105L, A117V, Y145X, Q160X, F198S, Q217R, Y218N, Y226X and Q227X [28,29,57]. Y218N demonstrated clinical criteria for possible Alzheimer disease and possible frontotemporal dementia, with neurofibrillary degeneration on neuropathological profiles, and various PrP^Sc ^fragments in the range of 10-80 kDa [58]. In general, neuropathologic features of GSS patients include numerous amyloid plaques, severe to absent spongiform changes, neuronal loss, astrocytic microgliosis, and variable nuerofibrillary tangles [28,29,59].

### Iatrogenic CJD (iCJD)

Iatrogenic CJD (iCJD) was first described in 1974 in a person who received cadaveric corneal transplant from a patient of CJD [22]. Several cases of human prion disease, since then, have been associated with iatrogenic transmission of CJD by the use of stereotactic intracerebral EEG needles or neurosurgical instruments, cadaveric dura mater grafts and intramuscular injections of contaminated cadaveric pituitary-derived human growth hormone (hGH) and gonadotrophin hormone. The highest proportion of iCJD cases is attributed to treatment with hGH and Lyodura grafts produced by B. Braun Melsungen AG of Germany and processed before May 1987. Most of CJD cases linked to treatment with hGH have occurred in France, while those linked to treatment with dura mater grafts have mainly occurred in Japan [28,29,51,60]. Between the late 1950s and 1985, about 30,000 children were treated with hGH worldwide, and the overall proportion of CJD cases from the treated population has been estimated to be about 1/100. It is believed that the likely PrP^Sc ^contamination of pituitary-derived hGH and dura mater grafts resulted from the practice of batch processing. Before the production of recombinant hGH, it was a pharmaceutical practice to process 5,000-20,000 cadaveric pituitary glands in a single batch for the extraction of the hormone. The presence in the batch of a few pituitary glands from unknown CJD cases may be responsible for contamination. With the availability of recombinant hGH and the initiation of separated processing of individual dura mater grafts since 1987, the probability of occurring future iCJD cases appears to be very low. A peak in the incidence of iCJD cases with long incubation periods may occur, however [28,29,60].

The clinicopathological features of CJD linked to treatment with hGH resemble with those of kuru. The 129 MM homozygosity is a risk factor and incubation periods may be in the range of 4.5 to over 25 years with a mean of 12 years [28,29,60,61]. In contrast, the clinicopathological features of CJD linked to neurografting of dura mater or the use of neurosurgical instruments resemble with those of sCJD. The mean duration of illness is 18 months and the incubation periods may be in the range of 1.5-18 years with a mean of about 6 years. In Japan, the minimum risk of developing iCJD has been estimated to be approximately 1/3000 Lyodura graft recipients [28,29,60].

### Kuru

Kuru is the first human prion disease that was shown to be transmissible to chimpanzees by intracerebral introduction of brain homogenates from kuru patients [62]. Kuru has occurred exclusively in the Fore linguistic group of Papua New Guinea Eastern Highlands and the neighboring peoples with whom they intermarried. There was a practice among these groups to consume the dead bodies of their relatives as a mark of respect and mourning (ritualistic cannibalism). Women and young children of both sexes were more exposed to the risk material such as brain and viscera than adult men who usually had preferentially to consume muscles. The kuru epidemic killed 1-2% of the population at its peak. Some villages became even devoid of adult women. With a ban on ritualistic cannibalism in the mid-1950s imposed by Australian authorities, the incidence of the disease started to decline steadily. Kuru was introduced to Western medicine in late 1950s, although the first case of kuru was observed around 1920. The Western scientists were very soon able to prove ritualistic cannibalism as the etiology of the disease. The older kuru patients who experienced an exposure to the infection before the ban can still be seen. The incubation periods of kuru in such patients would be more than 50 years [27,59,63-65]. Kuru has imposed strong selection pressure in the affected Fore groups on *PRNP *especially at codons 127 and 129. Heterozygosity at these *PRNP *codons is a resistance factor for kuru and can be seen with marked prevalence among survivors of the kuru epidemic [27,65].

Kuru has 3 clinical stages namely ambulant (still can walk), sedentary (only can sit up), and terminal (unable to sit up independently). An ill-defined prodromal period characterized by headache and pain usually in the joints of legs may precede these stages. Cerebellar ataxia, tremors and choreiform and athetoid movements are distinctive and prominent clinical signs. Shivering amplifiable by cold was the symptom on the basis of which the disease was named "kuru". The most prominent clinical feature of sCJD, the dementic illness can also occur in some cases but it happens during the final disease stages only. Neuropathological features such as spongiosis, neuronal loss, and astrocytic microgliosis can variably be observed in CNS usually in the grey matter. The PrP^Sc ^deposition is observed in CNS only [59,63]. The neuropathological property which distinguishes kuru from sCJD is the presence of numerous kuru plaques, spherical bodies with a rim of radiating filaments. Kuru is believed to be caused by the consumption of a sCJD case and experimental transmission studies have shown similarity between the molecular and pathobiological properties of prions causing kuru, sCJD and iCJD [66]. Similar studies have also revealed differences in transmission dynamics, peripheral pathogenesis and the neuropathology properties of kuru and vCJD prions. As both kuru and vCJD are caused by infection via oral route, these differences can be attributed to the strain type rather than the route of infection [63]. However, a recent study showed that sCJD, BSE and scrapie agents were evidently different from the kuru agent in incubation time, brain neuropathology, and the lymphoreticular involvement. The authors concluded that "the geographic independence of the kuru agent provides additional reasons to explore causal environmental pathogens in these infectious neurodegenerative diseases" [67,68].

### Variant CJD (vCJD)

The first 10 patients of new variant or simply of variant CJD (nvCJD/vCJD) were reported in April 1996 in UK [24,60]. The age of these patients was relatively younger between 16 and 39 years and they manifested a predominance of psychiatric symptoms instead of cerebellar ataxia or progressive dementia [60]. About 219 vCJD cases have been reported hitherto from 8 European and 4 non-European countries (USA, Canada, Saudi Arabia, and Japan). The most of vCJD cases (172) have been reported from UK alone [69]. Psychiatric and behavioral symptoms of vCJD may include agitation, aggression, depression, anxiety, apathy, emotional lability, insomnia, poor concentration, paranoid delusion, recklessness, or withdrawal; a combination of two or more of these symptoms appears in most of the patients. Some patients may also show signs of sensory disturbance such as pain, paresthesia, and dysesthesia. The neurologic symptoms occur at least 6 months after the onset of psychiatric symptoms and include cerebellar ataxia, cognitive impairment, involuntary movements which may be dystonic, choreiform or myoclonic. Incontinence of urine, progressive immobility, and akinetic mutism are the late onset signs. Death often occurs because of intercurrent infections. The mean age at onset of symptoms is 29 years, and the progression or total duration of the disease spans 18 months in average, which is similar to that is reported for kuru and iCJD linked to treatment with hGH [28,29,60]. Generalized slowing of EEG activity and an increase in the levels of 14-3-3 protein in CSF may be diagnostic of vCJD in more than 50% of the cases. However, the observation of posterior thalamic high signals characterized by 'pulvinar' or 'hockey stick' signs on T2 diffusion or Flair weighted MRI imaging and the detection of PrP^res ^in tonsillar biopsy are important for the specific diagnosis of the disease [29,70].

The neuropathalogical hallmark of vCJD is the presence of kuru-type amyloid plaques surrounded by spongiform lesions "the florid plaques" which also have been seen in scrapie but never in other human prion diseases [28,29,60]. Spongiform changes occur most evidently in the basal ganglia and thalamus with sparse distribution throughout the cerebral cortex. The florid plaques and PrP^res ^immunohistochemical staining have occurred predominantly in the cerebellum and cerebrum [28]. However, sufficient amounts of PrP^res ^are detected in the lymphoreticular system of vCJD patients [60]. The detection of PrP^res ^in the lymphoreticular system has confidently been used for the diagnosis of vCJD even in clinically normal subjects (carriers) [71]. To date, all vCJD patients have been 129 MM homozygotes [72].

Very soon after the report on 10 vCJD cases, epidemiological studies, experimental transmission of the disease to cynomologous macaques and mice (wild as well as transgenic) and biochemical strain typing linked the etiology of vCJD to infection from BSE prions [60]. The same or diverse phenotypes may develop on primary and secondary transmission of vCJD and BSE to mice expressing human PrP, depending on the source of inoculum and the PrP sequence of the recipient, and the 129 MV heterozygotes may be less resistant to vCJD transmission as compared to BSE transmission [73,74]. The E219K heterozygosity which has been considered a risk factor for transmission of BSE to humans may confer resistance to vCJD transmission within humans [70,75]. Moreover, at least 4 pathologically confirmed cases including 1 with subclinical or potentially preclinical infection have been associated with the secondary vCJD transmission via blood transfusion [76]. These reports on secondary iatrogenic transmission and the possibility of vCJD occurrence with long incubation periods in individuals with 129 MV and 129 VV PrP genotypes have raised serious public health concerns [71,72,77,78]. Some cases of kuru and iCJD have occurred after incubating the disease for almost 50 and 30 years, respectively [63,64,76,79].

### Variably protease-sensitive prionopathy (VPSPr)

In 2008, a novel form of atypical dementia was demonstrated in 11 patients, all of whom were 129 VV homozygotes and most of whom revealed a positive family history for cognitive impairment. No mutation in *PRNP *additional to the M129V polymorphism was detected. The clinical course of the disease was 2 years longer than sCJD but neuropathological profiles were diagnostic of TSEs. A more distinctive feature based on which the disease was named initially as "protease-sensitive prionopathy" (PSPr) was the reduced resistance of PrP^Dis ^isoforms to proteolysis by PK. The proteolysis of PrP^Dis ^isoforms by PK resulted in more than 3 PrP^Dis ^fragments. Amplification by PMCA and transmissibility to animal models of PrP^Dis ^yet remain to be investigated [26,42]. These studies will also inform on molecular mechanisms of the disease pathogenesis.

Similar to sCJD, PSPr can be seen in individuals harboring any of 3 M129V genotypes (129 MM, 129 MV and 129 VV). As the sensitivity of PrP^Dis ^isoforms to treatment with PK was variable according to the codon 129 genotype, the disease was renamed as "variably protease-sensitive prionopathy" (VPSPr). The PrP^Dis ^from 129 VV cases was more sensitive to PK treatment than was PrP^Dis ^from 129 MM cases, with PrP^Dis ^from 129 MV cases showing intermediary PK sensitivity [80]. Moreover, based on the codon 129 genotype, the PrP^Dis ^conformers from VPSPr cases not only showed differential immunoreativity to monoclonal antibodies raised against various PrP epitopes but also varied in the intensity (ratios) of individual fragments. Whether the codon 129 genotypes are associated with distinct clinical phenotypes, neuropathological lesions and histological distribution of PrP^Dis ^remains to be elucidated. To date, 30 cases with VPSPr including 20 of 129 VV, 7 of 129 MV and 3 of 129 MM *PRNP *genotype have been reported from various countries [42,80-82]. According to this data, the *PRNP *genotype 129 MM appears protective against the development of VPSPr [42].

## Conclusion

The epidemics of kuru, iatrogenic CJD and vCJD have reached their end [83]. However, sporadic and genetic cases of human prion diseases are occurring worldwide with an annual prevalence of 1-2 cases per million of population. Among genetic human prion diseases, GSS presents predominantly as an amyloidopathy. There is no cure for these disorders. Most of developing countries including Pakistan have no epidemiological information on human prion diseases. We have recently performed the analysis of *PRNP *in 909 unrelated Pakistani individuals. An insertion of 3 octapeptide repeats (3-OPRI) was found in heterozygous state in an individual and the extent of homozygosity at the 129 codon was 63%. These results indicate that Pakistani population is susceptible to prion disorders [84]. The octapeptide repeats region of human *PRNP *consists of 5 repeats which are designated as R1 through R5. If any nucleotide variation is found within a repeat, the name of that nucleotide is added to the name of corresponding repeat i.e., R2a or R3g [85]. A 3-OPRI (R1, R2, R2, R3g, R2, R2, R3, R4) linked to 219 K allele in one person (68 years old) and to 129 V allele in another (69 years old) has been associated with f/gCJD [86,87]. A 3-OPRI (R1, R2, R2, R2a, R2, R2, R3, R4) linked to 129 M allele has also been detected in a clinically normal Chinese young girl and her mother who was in her late 30's [85].

## Abbreviations

*PRNP*: Prion protein gene; PrP: Prion protein; PrP^C^: Cellular isoform of prion protein; PrP^Sc^: Scrapie isoform of prion protein; PrP^M^: Mutant PrP; TSE(s): Transmissible spongiform encephalopathy(ies); CJD: Cruetzfeldt-Jacob disease; sCJD: Sporadic CJD; vCJD: Variant CJD; f/gCJD: Familial/genetic CJD; iCJD: Iatrogenic CJD; FFI: Fatal familial insomnia; GSS: Gertsmann-Straussler-Scheinker syndrome; sFI: Sporadic fatal insomnia; PSPr: Protease-sensitive prionopathy; VPSPr: Variably protease-sensitive prionopathy; TME: Transmissible mink encephalopathy; CWD: Chronic wasting disease; BSE: Bovine spongiform encephalopathy; EUE: Exotic ungulate spongiform encephalopathy; FSE: Feline spongiform encephalopathy; NHP: TSE in non-human primates; OPR: Octapeptide repeats; OPRI: Insertion of octapeptide repeats; OPRD: Deletion of octapeptide repeats; GPI: Glycosylphosphatidylinositol; CNS: Central nervous system; EEG: Electroencephalography; CSF: Cerebrospinal fluid; CT: Computer tomography; MRI: Magnetic resonance imaging; PET: Positron emission tomography; PK: Proteinase K; cDNA: Complementary DNA; hGH: Human growth hormone; res: Resistant; Dis: Disease; kb: Kilo bases; kDa: Kilo Daltons; aa: Amino acids; A: Alanine; D: Aspartic acid; E: Glutamic acid; F: Phenylalanine; G: Glysine; H: Histidine; I: Isoleucine; K: Lysine; L: Leucine; M: Methionine; N: Asparagine; P: Proline; Q: Glutamine; R: Arginine; S: Serine; T: Threonine; V: Valine; W: Tryptophan; X: Stop codon; Y: Tyrosine.

## Competing interests

The authors declare that they have no competing interests.

## Authors' contributions

MI^1 ^surveyed literature and wrote the article. SM^2 ^organized the contents and submitted the article. All authors read and approved the final manuscript.
